# Modeling energy depletion in rat livers using Nash equilibrium metabolic pathway analysis

**DOI:** 10.1038/s41598-022-06966-2

**Published:** 2022-03-03

**Authors:** Angelo Lucia, Emily Ferrarese, Korkut Uygun

**Affiliations:** 1 Department of Chemical Engineering, University of Rhode Island, Kington, RI 02881 USA; 2 Center for Engineering in Medicine and Surgery, Massachusetts General Hospital, Boston, MA 02114 USA

**Keywords:** Systems biology, Diseases, Engineering

## Abstract

The current gold standard of Static Cold Storage (SCS), which is static cold storage on ice (about + 4 °C) in a specialized media such as the University of Wisconsin solution (UW), limits storage to few hours for vascular and metabolically active tissues such as the liver and the heart. The liver is arguably the pinnacle of metabolism in human body and therefore metabolic pathway analysis immediately becomes very relevant. In this article, a Nash Equilibrium (NE) approach, which is a first principles approach, is used to model and simulate the static cold storage and warm ischemia of a proposed model of liver cells. Simulations of energy depletion in the liver in static cold storage measured by ATP content and energy charge are presented along with comparisons to experimental data. In addition, conversion of Nash Equilibrium iterations to time are described along with an uncertainty analysis for the parameters in the model. Results in this work show that the Nash Equilibrium approach provides a good match to experimental data for energy depletion and that the uncertainty in model parameters is very small with percent variances less than 0.1%.

## Introduction

**Figure 1:**
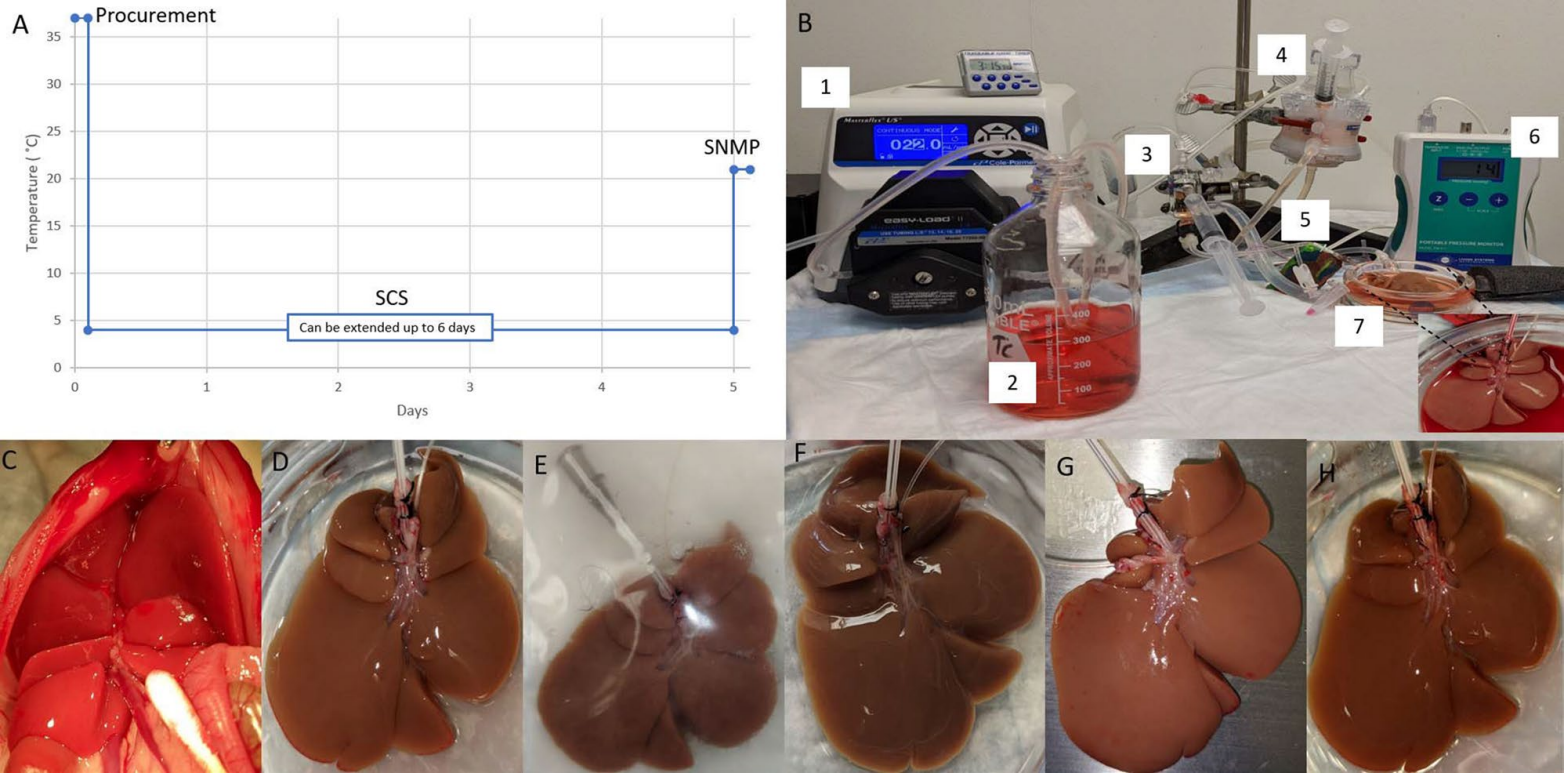
Overview of rat liver procurement, hypothermic preservation, and machine perfusion. (**A**) Temperature profile of static cold storage (SCS), Sub-Normothermic Machine Perfusion (SNMP), (**B**) Perfusion system 1. peristaltic pump, 2. media, 3. bubble trap, 4. membrane oxygenator, 5. pressure transducer, 6. pressure monitor, 7. organ basin; Steps in liver recovery, storage and perfusion starting with (**C**) liver in situ pre-flush, (**D**) procured and on ice post-flush, (**E**) at 24 h cold storage in UW solution, (**F**) Post lactated ringers flush, (**G**) starting SNMP, (**H**) end SNMP 3 h.

There are about 16,000 patients on the organ transplant waiting list in the US^1^, a number that far exceeds the supply of available organs. The current gold standard of Static Cold Storage (SCS), which is static cold storage on ice (about + 4 °C) in a specialized media such as the University of Wisconsin solution (UW), limits storage to few hours for vascular and metabolically active tissues such as the liver and the heart. This rather restrictive limit creates major logistical constraints that compound donor organ shortage and presents a major obstacle to on-demand tissue availability and global organ sharing^2^. There is general consensus in the field that traditional SCS is limited in part due to the slower but consensus depletion of the tissue energy stores which is a key upstream component of ischemia reperfusion injury; and our group has shown in a small clinical study that energy charge (a normalized combination of ATP, ADP, and AMP) is a marker of early allograft dysfunction for liver transplantation^3^. Given the limitations of SCS, the field itself is pushing ex vivo Machine Perfusion (MP) as an enabling technology compared to static cold storage (see Fig. 1 for a brief outline of a typical perfusion protocol). In the last two decades, efforts from several groups^4–8^ have focused on extracorporeal machine perfusion to enable transplantation of currently discarded ischemic organs, culminating in randomized trials which report safety^9^ and dramatically increased utilization of donor livers with machine perfusion^10^.

Despite such efforts, there has never been a rational design of the preservation media based on systems engineering principles, to take full advantage of the machine perfusion to manipulate the organ metabolism fully to recover from ischemic injury^11^. This, in turn, could allow significantly increasing the donor organ pool, by gainfully utilizing the currently discarded organs.

The problem of liver preservation has several unique properties that simultaneously help and hinder utilizing systems engineering approaches for optimizing the organ. The liver itself is arguably the pinnacle of metabolism in human body, and therefore various metabolomic pathway analysis approaches immediately become very relevant. The prior elucidation of central energy metabolism as discussed above, and the finding of energy charge as a clinical biomarker^3^ further brings central energy metabolism into focus. There is also extensive prior work on liver metabolic flux analysis (Yang et al.^12^, Lee et al.^13^, Uygun et al.^14^, Sharma et al.^15^, Orman et al.^16,17^, and others) including comprehensive stoichiometric models such as hepatonet1 (Gille et al.^18^). Moreover, in the context of transplantation, preservation is a process of just a few hours, meaning many genetic aspects can be safely neglected provided the SNMP temperature is less than 10 °C. In the absence of blood, immune responses are subdued which makes this otherwise key aspect of liver biology likely safe to ignore, at least initially in model development. Finally, preservation allows easy access to sampling both the tissue and the preservation media for analysis, allowing obtaining time profile data with significant ease compared to typical medical applications.

On balance though, this is a difficult problem. Approaches targeting pseudo steady-state, such as Metabolic Flux Analysis (MFA; an experimental technique used to quantify production and consumption of metabolites and cofactors, see ^12,13^) or Flux Balance Analysis (FBA; see Varma and Palsson^19^ for an introduction to the basic concepts of FBA and Smallbone and Simeonidis^20^ for a geometric interpretation) and many of its variations are by definition limited. The dynamic FBA (dFBA) approach of Mahadevan et al.^21^ and similar approaches are more applicable, but the majority of FBA-based approaches are constrained only by reaction stoichiometry, which often results in degenerate solutions that lie on user-specified flux bounds^22^. Dynamic formulations that incorporate chemical reaction kinetics (see Zielinski and Palsson^23^), which can be traced back to the work of Michaelis and Menten^24^ are ideal but suffer from a need of experimentally determined kinetic parameters which grow exponentially with the model size, are not practical in the context of an organ.

In this work, we propose a novel approach for modeling and numerical simulation of liver metabolism during static cold storage and warm ischemia (WI) that leverages the Nash Equilibrium (NE) methodology recently developed by Lucia and coworkers^25–27^ and enables modeling the dynamics with a very limited set of thermodynamic parameters that are readily accessible in literature. A general overview of the NE methodology is presented, followed by a description of the generalized metabolic network model developed for NE modeling of perfusion, in turn followed by numerical results for Nash Equilibrium (NE) simulations of liver metabolism for SCS and WI. Model predictions, comparisons to literature data, as well as algorithm convergence, and an uncertainty analysis of model parameters are discussed. We end the paper with a discussion of key results and conclusion of this work.

## Materials and methods

### Nash equilibrium modeling

The modeling approach used in this manuscript is based on the Nash equilibrium (NE) framework for determining unknown fluxes throughout a given network recently developed by Lucia and coworkers^25–27^ and is quite unlike FBA, MFA, or kinetic modeling. The NE approach is a first principles approach that uses chemical equilibrium and mass balance and is based on the following three ideas:

1. Enzymes are players in a multi-player game.
2. Each player (enzyme) minimizes the Gibbs free energy of the reaction it catalyzes subject to element mass balances.
3. The goal of the metabolic network is to find the best overall solution given the natural competition for nutrients among enzymes.

A detailed description of the mathematical framework associated with the Nash Equilibrium approach is given in Lucia and DiMaggio^27^, the first part of which is a tutorial describing the mathematical formulation, governing equations, and algorithmic aspects such as charge balancing, linear independence of constraints, enzyme–substrate reactions, etc. Here we present the basic formulation as well as a Nash Equilibrium pseudo-algorithm in order to give the reader a better overall understanding of the sequence of computations used in the general case.

### Formulation

The NE formulation for an arbitrary metabolic network is a rigorous, first principles approach that does not ignore or constrain accumulation/depletion of intermediate metabolites and cofactors.

The NE formulation is given by a collection of $j = 1,2, \ldots ,N$ nonlinear programming (NLP) sub-problems of the form

1 $$\left[ {minimize\frac{{G_{j} \left( {\nu_{j} } \right)}}{RT}subject \, to \, conservation \, of \, mass, \, v_{j}^{*} } \right]$$

where $\frac{{G_{j} }}{RT}$, the dimensionless Gibbs free energy, is the objective function associated with the appropriate enzyme that catalyzes one or more reactions at a given node $j$ in the network, $R$ is the universal gas constant, and $T$ is absolute temperature. The conservation of mass constraints are elemental mass balances that often involve charged species and, as in other approaches, $\nu_{j}$ represents the fluxes of metabolic material at node $j$. Finally, the vector, $\nu_{ - j}^{*}$, denotes the optimal fluxes of all other sub-problems, $k = 1,2, \ldots ,j - 1,\,\,j + 1, \ldots ,N$. The Gibbs free energy for sub-problem $j$ is given by

2 $$\frac{{G_{j} }}{RT} = \mathop \sum \limits_{i = 1}^{{C_{j} }} x_{ij} \left[ {\frac{{\Delta G_{ij}^{0} }}{RT} + \ln \left( {\mu_{ij} } \right)} \right]$$

where $\Delta G_{ij}^{0}$ are the standard Gibbs free energies of reaction at 25 °C for the metabolic reactions associated with sub-problem $j$, $x_{ij}$ are mole fractions, which are calculated from the fluxes, $\mu_{ij}$ are chemical potentials, $i$ is a component index, and $C_{j}$ is the number of components associated with a sub-problem $j$ in the network.

Temperature effects are included using the Gibbs–Helmholtz equation, which is given by

3 $$\frac{{\Delta G_{ij}^{0} \left( T \right)}}{RT} = \frac{{\Delta G_{ij}^{0} \left( {T_{0} } \right)}}{{RT_{0} }} + \frac{{\Delta H_{ij}^{0} \left( {T_{0} } \right)}}{R}\left[ {\frac{{\left( {T - T_{0} } \right)}}{{TT_{0} }}} \right]$$

where $T_{0}$ is the reference temperature (usually 298 K), $T$ is the temperature at which the reaction takes place (say 310  K ), and $\Delta H_{ij}^{0} \left( {T_{0} } \right)$ is the standard enthalpy change of reaction $i$ at node $j$ in the network. All standard Gibbs free energy changes due to reaction,$\Delta G_{ij}^{R0} \left( {T_{0} } \right)$, and the enthalpy changes due to reaction, $\Delta H_{ij}^{R0} \left( {T_{0} } \right)$, can be computed from Gibbs free energies and enthalpies of formation and reaction stoichiometry. For example,

4 $$\Delta G_{ij}^{R0} = \mathop \sum \limits_{k = 1}^{{n_{p} \left( {i,j} \right)}} S_{k} \Delta G_{f,ijk}^{0} - \mathop \sum \limits_{k = 1}^{{n_{r} \left( {i,j} \right)}} S_{k} \Delta G_{f,ijk}^{0}$$

where the $S_{k}$'s are the actual stoichiometric numbers based on conversion and $n_{p} \left( {i,j} \right)$ and $n_{r} \left( {i,j} \right)$ are the number of products and number of reactants respectively associated with reaction $i$ and node $j$.

All necessary Gibbs free energies of formation data has been assembled in a local database and was taken from eQuilibrator (http://equilibrator.weizmann.ac.il/). Feedbacks within any pathway are converged using successive substitution. Thus, issues of thermodynamics infeasibility raised in Henry et al.^28^ are irrelevant in the NE framework. Also, any number of enzyme–substrate reactions can be explicitly included in the model and require.

1. The RCSB Protein Data Bank (https://www.rcsb.org/pdb/home/) for accurate enzyme and/or enzyme–substrate structure data,
2. AutoDock or 1-Click-Docking (https://mcule.com) to compute the necessary Gibbs free energies of binding (or binding affinities) for all key enzymes.

### Nash equilibrium pseudo-algorithm

The Nash Equilibrium algorithm is iterative.

1. Define a metabolic network.
2. Identify all pathways (and species: metabolites, cofactors, enzymes), pathway connections, and transport fluxes in the given metabolic network.
3. Read properties for all species (i.e., Gibbs free energies and heats of formation, molecular weights, etc.).
4. Break all feedback and transport fluxes in the network and estimate the initial values of these fluxes.
5. Determine an initial steady-state solution.
6. Perturb the metabolic network to elicit a response.
7. Solve each NLP sub-problem given by Eqs. (1–4) for the minimum Gibbs free energy for all unknown fluxes, including feedback and transport fluxes.
8. Compare the new calculated transport fluxes with previously estimated flux values.
  - If the 2-norm of the difference is within a certain tolerance, $\epsilon= 10^{ - 3}$, stop.
  - If not, replace estimated feedback and transport fluxes with the calculated ones; go to step 7.

Steps 1–5 are preprocessing steps. Step 1 defines the metabolic network to be simulated. Step 2 creates link-lists of pathways, species, and transport fluxes. Each pathway in the given network has well-defined reactions and thus all species, charged and/or neutral, can be identified. Given the identities of all species, step 3 reads the appropriate data from our database. Because the NLP sub-problems to be solved are nonlinear and feedback and transport fluxes exist, all feedback and transport fluxes must be estimated initially. This is done in step 4 of the pseudo-algorithm. Step 5 determines an initial steady-state solution from which the network is perturbed in step 6 to elicit a response. The software is designed to permit perturbation of any metabolite, cofactor, or enzyme and to add/delete any pathway from the network. Step 7 is the heart of the NE approach in which all NLP sub-problems are solved for the unknown fluxes of all metabolites, cofactors, and enzymes, including feedback and transport fluxes. It is important to note that there may (and most likely will) be multiple fluxes for many of the common cofactors in metabolism. For example, there will be an ATP flux in glycolysis as well as one for ATP in the Krebs cycle, the urea cycle, oxidative phosphorylation, and so on. Finally, in step 8 feedback and transport fluxes on successive NE iterations are compared. Step 8a checks for convergence and, if successful, the NE algorithm terminates. If convergence is not reached, step 8b is invoked (successive substitution), and steps 7 and 8 are repeated, which define the next NE iteration. Converged NE solutions provide values of all fluxes, concentrations, amounts of all metabolites, cofactors and enzymes generated and/or consumed in the model, pH, and energy charge.

### Modeling liver metabolism for organ preservation

**Figure 2:**
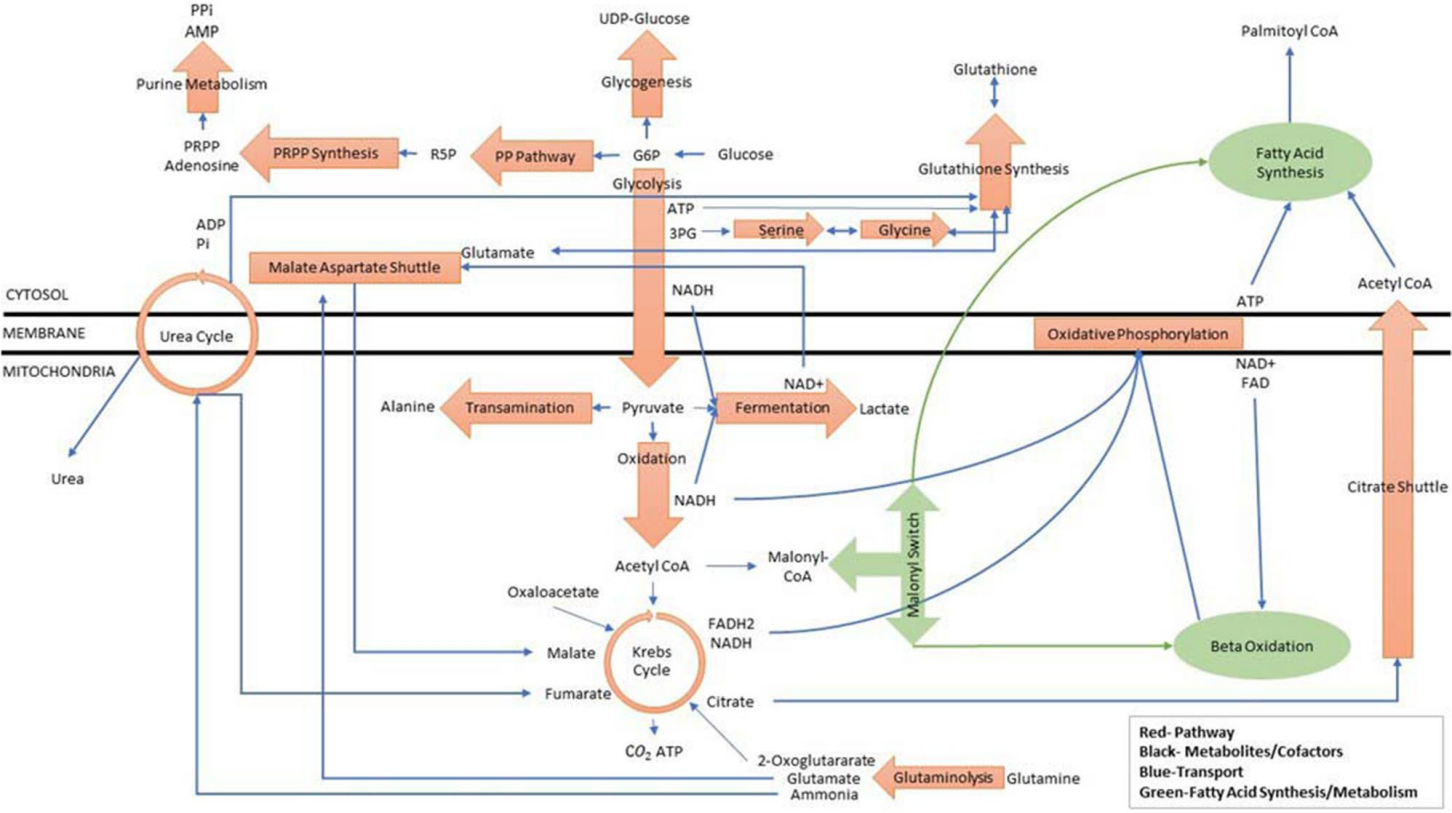
Superstructure representation of liver energy metabolism for study of preservation.

To study the central energy metabolism during liver preservation, we constructed a superstructure capturing the key pathways, as presented in Fig. 2.

The superstructure depicted in Fig. 2 is flexible and allows us to simulate liver metabolism in SCS, WI, and in the future MP in any number of ways, including without fatty acid synthesis or fatty acid metabolism (beta-oxidation), (2) fatty acid synthesis, no beta-oxidation, (3) beta-oxidation but no fatty acid synthesis, and (4) fatty acid synthesis and beta-oxidation. In this work, we consider only fatty acid metabolism. In addition, one of the key features of the Nash equilibrium approach is that it allows metabolic reactions to be driven in the forward or reverse direction. For example, interconversion of serine and glycine is reversible and can be used to either produce or consume glycine.

### Key aspects of liver metabolism incorporated in the model

There are a number of unique aspects of liver metabolism that should be included in order to have a physiologically meaningful mathematical model in the context of liver SCS and WI.

#### Glycolysis

Conversion of glucose to glucose-6-phosphate (G6P) is carried out by hexokinase 4 (glucokinase) which is only present in the liver and pancreas. Unlike other isoforms of hexokinase, the glucokinase catalyzed reaction

5 $${\text{Glucose}} + {\text{ATP}} = {\text{G6P}} + {\text{ADP}} + {\text{H}}^{ + }$$

is not inhibited by G6P but requires much higher glucose concentrations.

#### Glutathione Synthesis

Southard et al.^29^ report that glutathione, along with adenosine, are important components of the University of Wisconsin (UW) solution used in static cold storage organ preservation. These key nutrients help with ATP regeneration. To see this, note that high glutathione concentration will shift the overall reaction

6 $${\text{glycine}} + {\text{2ATP}} + {\text{glutamate}} + {\text{cysteine}} = {\text{2ADP}} + {\text{2Pi}} + {\text{glutathione}}$$

to the left, provided there is sufficient ADP and Pi available. This results in the production of the three amino acids (glycine, serine, and glutamate) and ATP and is accomplished by cleaving the cysteine-glycine dipeptide by a membrane peptidase enzyme. See Lu^30^.

#### Serine biosynthesis

Serine is generally produced from 3-phosphoglucose (3PG). However, the liver does not synthesize serine to any appreciable degree because the enzyme 3-phosphoglycerate dehydrogenase (3-PGDH) is only present in very small amounts. See Kalhan and Hanson^31^. This suggests that the liver transports very little, if any, 3PG from glycolysis to the serine biosynthesis pathway and must synthesize serine differently. The enzyme glycine hydroxymethyltransferase (SHMT2), which is present in mice, rats, and humans, can convert glycine synthesized from glutathione to serine.

#### Lactate

 Lactate is produced during SCS because the liver is in a hypoxic state and pyruvate metabolism shifts from oxidation to fermentation. Typical healthy concentrations of lactate range from 2 to 3 mM and represent one criterion commonly used to assess of liver viability during MP.

#### Ammonia and ammonium toxicity

Ammonia (NH_3_) is toxic to animals and human. Normal intracellular ammonia concentrations are in the range 0.5 to 1 mM. See Remesy et al.^32^. Ammonium (NH_4_^+^) is also toxic. Levels ~ 100 mmol can cause loss of consciousness and levels ~ 200 mmol can cause convulsions and/or coma. Thus, it is important to keep levels of ammonia and ammonium at acceptable levels in SCS and MP and in turn simulating them allows tracking these constraints on the organ.

### Static cold storage (SCS) simulations

All SCS simulations in this manuscript were run at 4 ͦC in order to mimic the storage of livers, which are flushed with UW solution at recovery and then placed on ice for transport to the recipient hospital. Table 1 shows the relative composition of the important components in the UW solution used in this study, as reported by Southard et al.^29^ (p. 252, column 2). In our simulations, we calculated the concentrations of these metabolites based on 50 ml. UW solution flush.

**Table 1:** UW solution components (@ 4 °C).

Compound	Amount (μmol/cell)	Southard et al.
Glutathione	0.3	3 mM
Adenosine	0.5	5 mM
Water	5.54	

It is important for the reader to understand that complete knowledge of the metabolic state at the beginning of SCS is not possible from experiment. To circumvent this, we compute a chemical equilibrium and use this as the initial state of the liver in SCS.

## Results

### Static cold storage simulation results and comparison with experimental data

**Figure 3:**
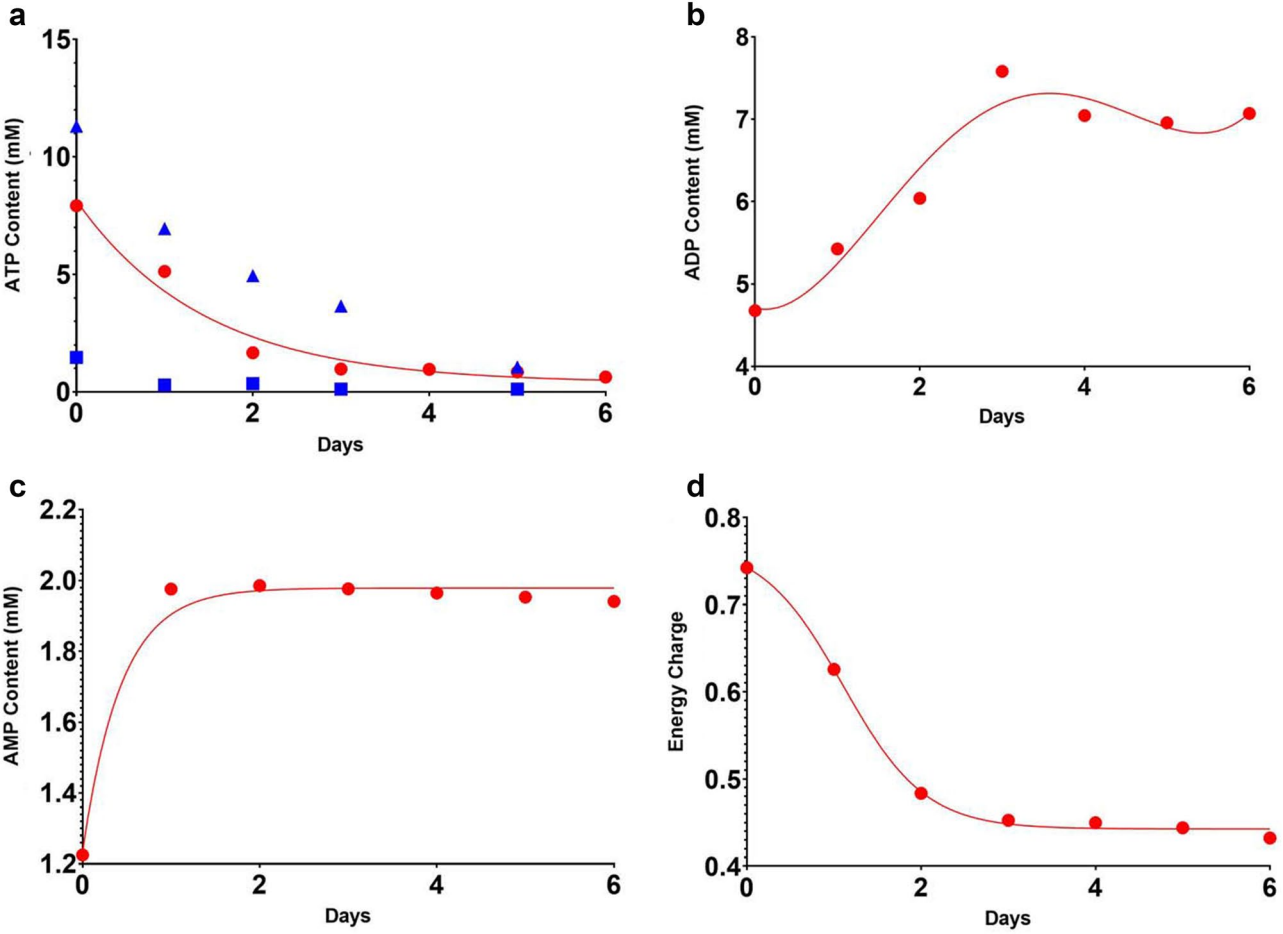
Nash equilibrium predictions of ATP, ADP, and AMP content (mM), and energy charge for static cold storage (SCS) as a Function of Storage Time: (**a**) ATP content (mM):  NE simulation iterations,  least-squares fit of NE iterations, Berendsen et al.^36^,  Bruinsma et al.^37^, (**b**) ADP content (mM), (**c**) AMP content (mM), (**d**) Energy charge.

**Figure 4:**
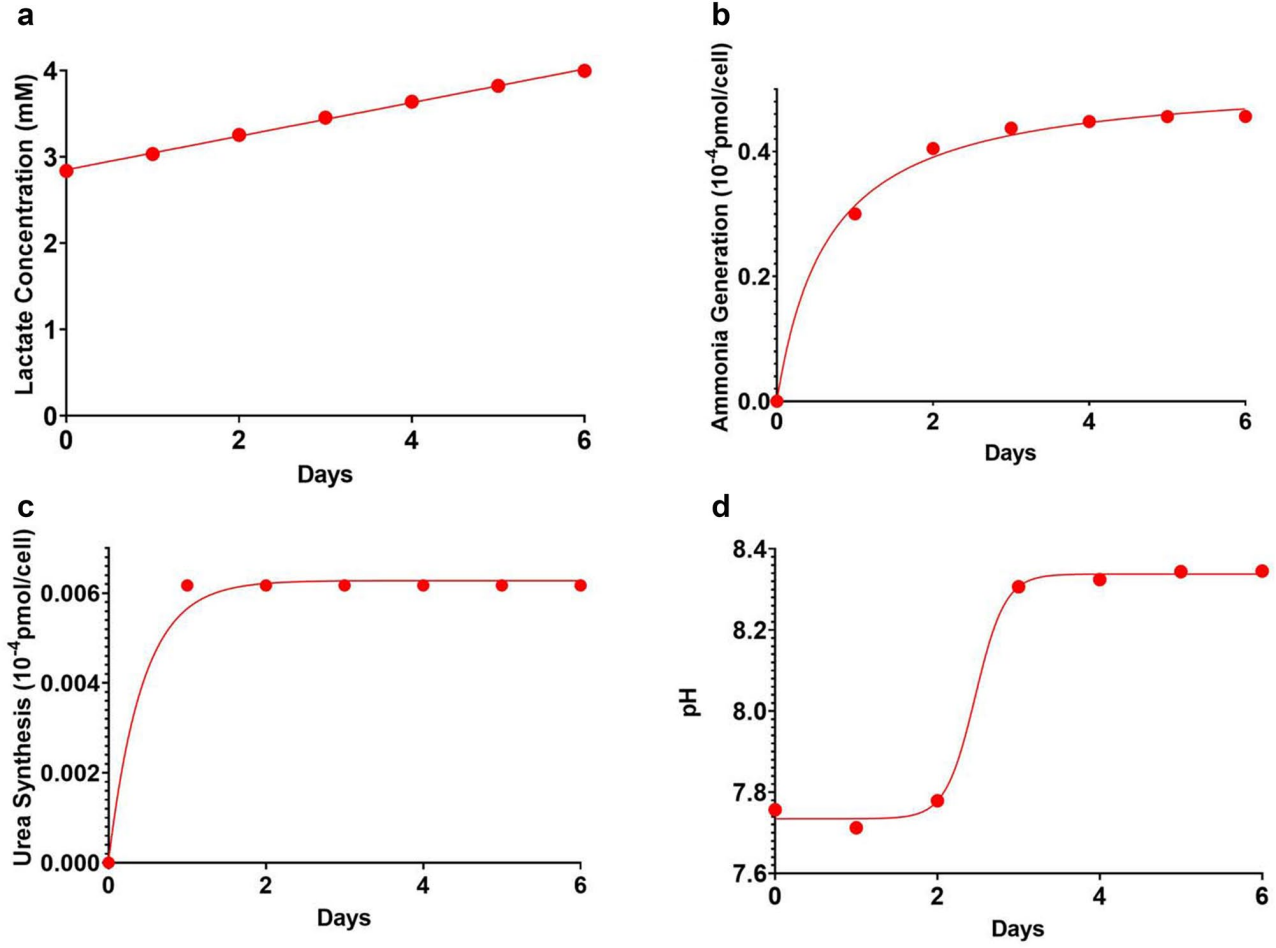
Time profile of select metabolites and pH during cold storage: (**a**) Lactate concentration, (**b**) Cumulative ammonia generation, (**c**) Cumulative urea synthesis, (**d**) pH.  NE simulation iterations,  least-squares fit of NE simulation iterations.

Numerical simulation results for static cold storage are shown in Figs. 3 and 4. Figure 3 shows the time evolution of ATP, ADP, and AMP content, as well as energy charge, which is a normalized ratio of the three energy carriers. Note the duration of the Nash Equilibrium computations required to reach steady-state actually takes six days—with a net depletion of ATP of 2.777 × 10^–4^ pmol/cell and a decrease in ATP content from 7.923 to 0.6343 mM. Over the same period, the energy charge decreases from 0.7422 to 0.4321 due to the depletion of ATP coupled with an increase of 2.5597 × 10^–5^ pmol/cell of AMP.

There is not a lot of experimental data for static cold storage in the open literature and the experimental data sets that are available show considerable variability, most likely due to the extreme sensitivity of ATP assays to sample gathering and freezing protocol. Figure 3a shows a comparison of the Nash Equilibrium predictions of ATP content in mM with the experimental data^33,34^, containing ATP content data from a total of 44 and 30 rats respectively, whose weight ranged from 150 – 250 g. In both studies the authors state that ATP content correlates well with liver graft viability. Note that while the ATP content for the two experimental data sets has considerable variability, the ATP concentration is in the normal range of [0.1, 10 mM] and both data sets follow similar time evolution. Notably, the model predictions fall between the two studies in literature and are within the 99% confidence interval of the work of Berendsen et al.^36^ and provides a good qualitative match of the experimental data. See “Appendix A” for the conversion of experimental data reported in pmol ATP/mg protein to concentration in mM.

### Behavior of other metabolites and cofactors during cold storage

Figure 4 shows the metabolic behavior of lactate concentration, total ammonia synthesis, cumulative urea synthesis, and pH in static cold storage.

Here the net amount of ammonia produced was 4.728 × 10^–5^ pmol/cell while the concentration of lactate concomitantly rose from 2.836 to 4.048 mM due to the synthesis of 4.148 × 10^–5^ pmol/cell of lactate. This is above the normal lactate concentration of 2–3 mM. The SCS numerical simulations also show that there was some ammonia clearance from the liver with the synthesis of 1.643 × 10^–6^ pmol/cell of urea resulting in a final ammonia concentration of 4.553 mM, which is considerably higher than the normal intracellular ammonia concentration of 0.5 to 1 mM. During SCS pH rose from 7.7567 to 8.3452 due to the consumption of H^+^ ions in pyruvate fermentation to produce lactate and in the electron transport chain. Other key metabolites that underwent change during static cold storage simulations included glutathione, NAD^+^, ADP, and water. Glutathione consumption helped offset depletion of ATP. Synthesis of NAD^+^ resulted in an NAD^+^/NADH = 2.6752, which is below the normal range. Finally, it is important not to overlook the consumption of intracellular water during SCS due to urea synthesis and the synthesis of ADP.

### Time evolution

**Figure 5:**
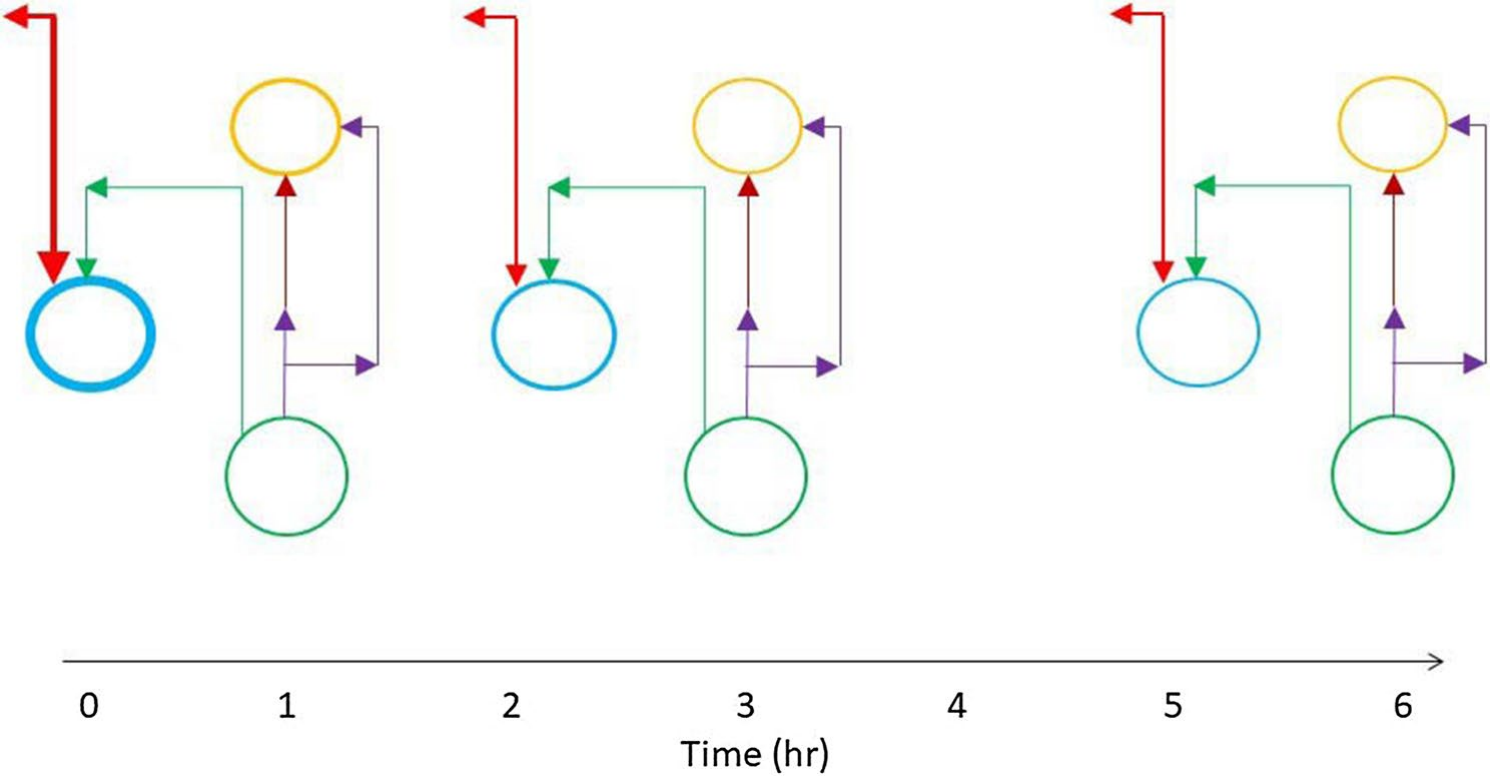
Snapshots of key transport fluxes as a function of time at days 1, 3 and 6 in static cold storage. Flux colors: green = TCA cycle, light blue = urea cycle, gold = fatty acid synthesis, red = glutaminolysis, purple = citrate shuttle, dark red = malonyl CoA. Higher fluxes are indicated by thicker lines.

The methodology to convert NE iterations to time is described in detail in “Appendix B”. Figure 5 gives snapshots in time of the behavior of key inter-pathway transport fluxes and dominant pathways, where the thicker lines indicate higher fluxes.

The urea cycle and fatty acid synthesis dominate the dynamic behavior of the network because these pathways are the major consumers of ATP. The flux of ammonia in glutaminolysis is high as are the inter-pathway transport fluxes of ammonia to the urea cycle and pyruvate to pyruvate fermentation – as shown by the thicker red arrows on the left of Fig. 5. As a result, these pathways are activated in order to remove ammonia and produce lactate respectively. In addition, the fluxes of acetyl-CoA and butanoyl-CoA are elevated in fatty acid synthesis. As time progresses and ATP is depleted the inter- and intra-pathway fluxes in the urea cycle, pyruvate fermentation, and fatty acid synthesis diminish but remain dominant.

### Model parameter uncertainty in static cold storage simulations

The main model parameters used in the Nash Equilibrium approach are standard state Gibbs free energies, $\Delta G_{f,i}^{0}$, and enthalpies, $\Delta H_{f,i}^{0}$, of formation. As noted earlier, all standard state Gibbs free energies of formation, $\Delta G_{f}^{0}$, were taken from the eQuilibrator database (http://equilibrator.weizmann.ac.il/), which also includes standard deviations, $s$. For example, the standard state Gibbs free energy of formation for NAD^+^ given in the eQuilibrator database is $\Delta G_{f,NAD + }^{0} = - 1171.80 \pm 12.4$ kJ/mol. For ATP, $\Delta G_{f,ATP}^{0} = - 2295.10 \pm 3.0$ kJ/mol. Standard deviations in the eQuilibrator database are quite small and range from 0 to ~ 20 kJ/mol or 0 to ~ 1.5%. For each simulation, all 78 values of $\Delta G_{f}^{0}$ were varied independently within their respective standard deviations using a separate random number in the range (0, 1] for each. One hundred (100) random samples (i.e., metabolic network simulations) starting from the same exact initial state were run.

**Figure 6:**
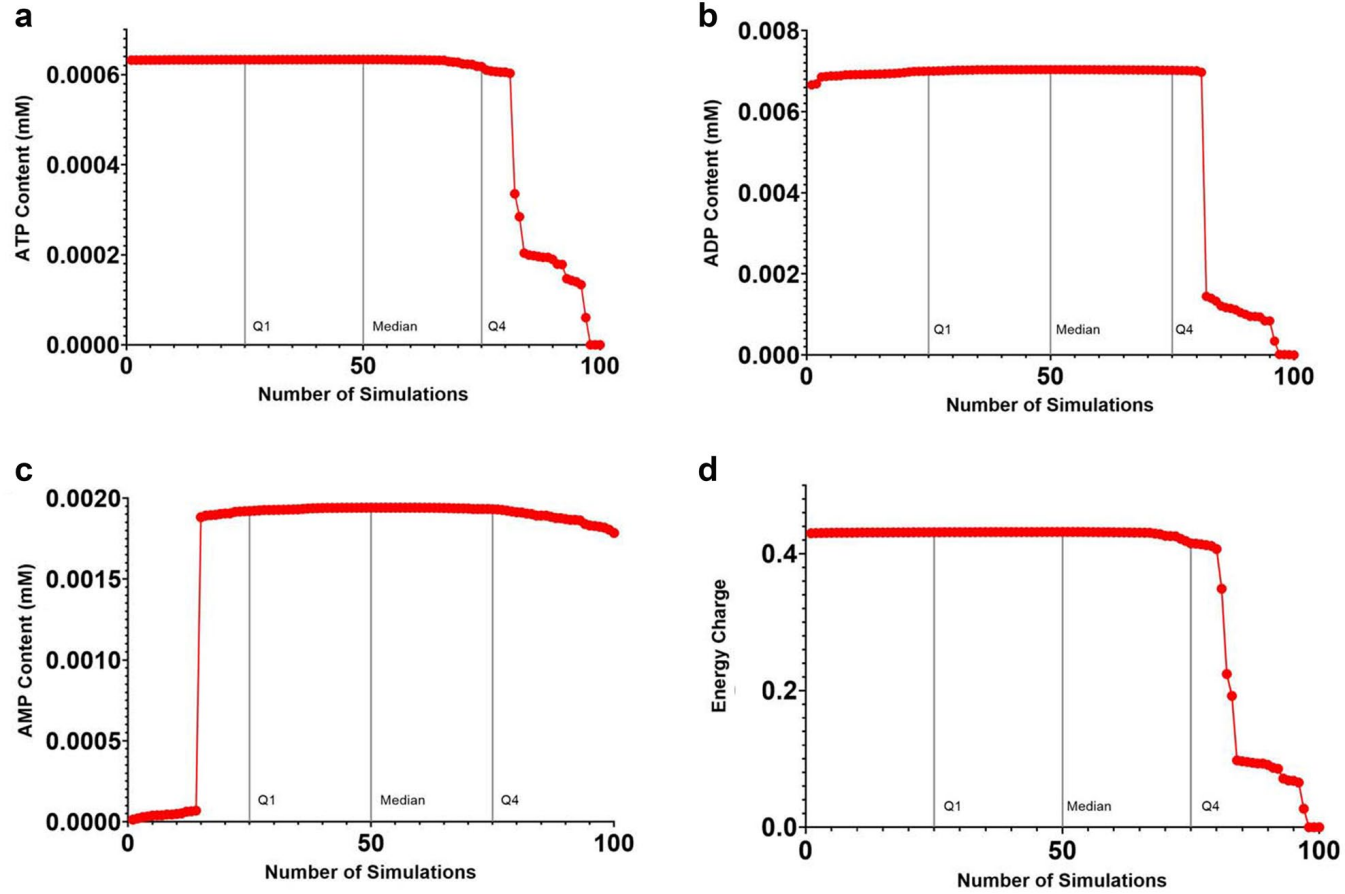
Parameter Uncertainty Probability Density Functions for 100 Static Cold Storage Simulations. (**a**) ATP content (mM), (**b**) ADP content (mM), (**c**) AMP content (mM), (**d**) Energy charge.

A mean value and variance, $\sigma^{2}$, for the amount of ATP, ADP, AMP, and energy charge were determined, along with values for lactate, ammonia, and urea concentration, as well as pH. Figure 6 shows the probability density function for ATP, ADP, and AMP in mM and energy charge for 100 SCS simulations. The values of $\Delta G_{f}^{0}$ used in this study are given in “Appendix C”.

All quantities in Fig. 6 are described by a generalized gamma distribution. Note that there are very small variances in ATP, ADP, AMP, and energy charge between the first interquartile, $Q_{1}$, and the fourth interquartile, $Q_{4}$, indicating that the liver model is very consistent and robust. Table 2 gives the statistics for the parameter uncertainty analysis. Note that the NE framework predicts very small variances for all metabolites and cofactors. In particular, the variance for ATP content is on the order of 10^–8^ while the percent variance is on the order 10^–6^ for 100 SCS simulations. The median value and interquartile range (IQR) for ATP content are 6.33595 × 10^–4^ mM and 1.54060 × 10^–5^ respectively.

**Table 2:** Parameter uncertainty analysis for liver energy metabolism in static cold storage.

Quantity	Average value	Variance	% Variance
ATP conc (mM)	0.73101	5.28047 × 10^–8^	7.22353 × 10^–6^
ADP conc (mM)	0.71249	4.72755 × 10^–8^	6.63525 × 10^–6^
AMP conc (mM)	1.93966	1.90869 × 10^–11^	9.84033 × 10^–8^
Lactate conc (mM)	4.05250	6.71574 × 10^–11^	1.65718 × 10^–10^
Ammonia conc (mM)	7.22165	4.07778 × 10^–12^	5.64660 × 10^–11^
Urea conc (mM)	0.12662	3.56513 × 10^–13^	28.1561 × 10^–10^
Energy charge	0.43775	1.68229 × 10^–4^	3.84303 × 10^–2^
pH	8.33438	6.54638 × 10^–3^	7.85466 × 10^–2^

100 random SCS metabolic network simulations initialized from a UW solution flush.

An IQR of 1.54060 × 10^–5^ means that 50% of the data points are within 1.54060 × 10^–5^ times the median consumption of ATP or in the range [6.17687 × 10^–4^, 6.33093 × 10^–4^]. The variances and percent variances for energy charge and pH are a bit larger than those for the metabolites and cofactors; however, all percent variances are less than 0.1%. The total time required for 100 SCS simulations was 30.96 s., giving an average liver metabolism SCS simulation time of ~ 0.31 s. Therefore, many SCS scenarios can be investigated very quickly using numerical simulation, something not feasible with experimentation.

### Nash equilibrium convergence behavior for static cold storage simulation

The convergence behavior of the Nash equilibrium algorithm involves two levels of iteration:

1. *Inner Loops* Iteration is required to sequentially converge individual pathways that contain feedback such as glutaminolysis, the Krebs cycle, oxidative phosphorylation, and the urea cycle. Within each outer loop iteration, pathways with feedback are converged to an accuracy of 10^–5^. Thus, at each outer loop iteration, all pathways are in (quasi-) chemical equilibrium.
2. *Outer Loop* Iteration is also needed to converge the outer loop variables which consist of inter-pathway transportation fluxes. See the blue lines in Fig. 2.The convergence criterion used here is defined in step 8 of the pseudo algorithm and in this study had a value of $\epsilon= 10^{ - 3}$.

Given this algorithmic structure, it is the behavior of the inter-pathway transport fluxes that dictate the overall time evolution of the network and convergence performance. Table 3 provides an example of the convergence behavior of a Nash Equilibrium simulation of static cold storage, which, in this instance, required only 0.36 CPU sec.

**Table 3:** Convergence behavior of NE simulation of static cold storage.

Time (days)	2-norm of transport fluxes
1	0.38375
2	0.16993
3	0.07991
4	0.03381
5	0.03281
6	0.00038

### Warm ischemia (WI) simulation results and comparison with experimental data

**Figure 7:**
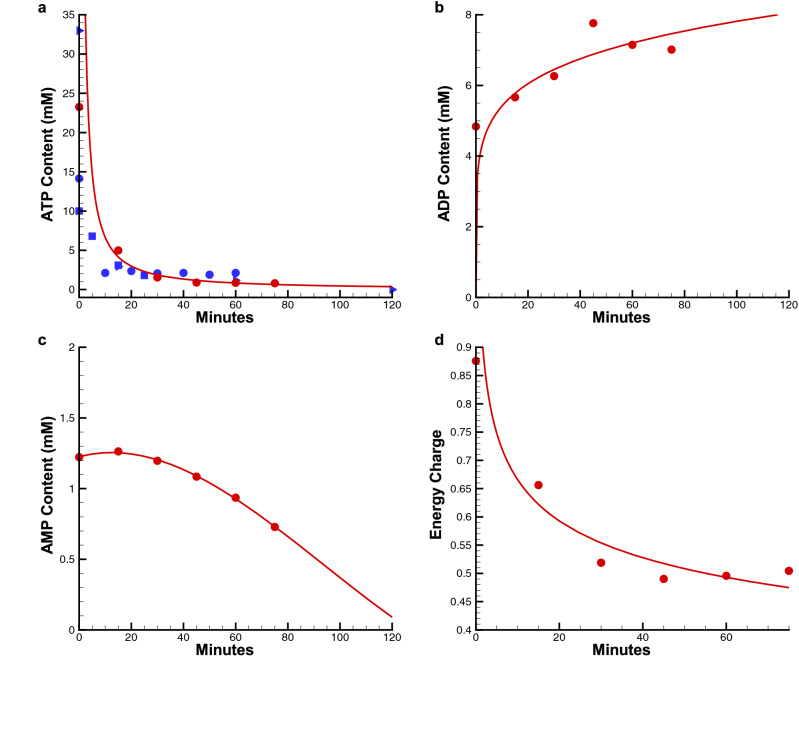
Nash equilibrium predictions of ATP, ADP, and AMP content (m), and energy charge for warm ischemia (WI) as a Function of Time: (**a**) ATP content (mM):  NE simulation iterations,  least-squares fit of NE iterations,  Kamiike et al.^39^ (rat liver),  Bore et al.^40^ (rat kidney),  Harrison et al.^41^ (rat heart), (**b**) ADP content (mM), (**c**) AMP content (mM), (**d**) Energy charge.

There are many studies of ATP depletion for liver, kidney, heart, etc. in rats, pigs, and humans in warm ischemia and all studies show basically the same qualitative behavior—a rapid decrease in cellular ATP content, usually within 20–30 min. To further validate the Nash Equilibrium modeling framework, several numerical simulations were run using the exact same model of liver metabolism shown in Fig. 2. To simulate warm ischemia, the temperature was set to 37 °C. Figure 7 shows representative NE simulation results for ATP depletion, along with ADP and AMP content as well as energy charge during warm ischemia and compares the ATP depletion predicted by simulation with experimental ATP depletion data^39–41^.

Note that the NE simulations agree with the experimental data quite well, capturing rapid ATP depletion within the first 20 min. However, it is important to note that while the pathways for WI were the same as those for SCS, the numerical behavior of glycogenolysis and glycolysis were markedly different. Most notably, the rate of glycogenolysis (i.e., the reverse of glycogenesis that produces glucose 1-phosphate (G1P) from glycogen) was much higher in warm ischemia than in SCS. See Harrison et al.^41^ This, in turn, significantly increased the rate of glucose 6-phosphate (G6P) synthesis and the rate of ATP consumption in the preparatory phase of glycolysis. As in Martin et al.^42^, we observed a reduction in NAD^+^ concentration and higher accumulation of glyceraldehyde-3-phosphate (G3P) in the warm ischemia NE simulations, both of which impact the payoff phase of glycolysis. As a result, net ATP production was slightly less in WI than in SCS while net ATP consumption was 2.5 times higher in WI than in SCS.

## Discussion and conclusions

A metabolic pathway model for rat livers was presented along with an algorithm for Nash Equilibrium (NE) modeling and simulating metabolic networks. Numerical results have demonstrated that the NE approach can be used to successfully model and simulate UW flush plus static cold storage as well as warm ischemia in rat livers. Numerical results also showed that the NE framework can produce numerical behavior of SCS that compares favorably with experimental data. That is, NE simulations of SCS showed a marked decrease in both ATP content and energy charge along with concomitant generation of lactate, ammonia, and urea. Snapshots of the time evolution of static cold storage were presented and indicate that fluxes in the urea cycle, pyruvate fermentation, and fatty acid synthesis are higher than in other parts of the network throughout the entire SCS simulation and dominate the time behavior of SCS. Moreover, this dynamic behavior is precisely what one would expect. Finally, NE simulations of warm ischemia were conducted. Here, numerical results were in good agreement with experimental data, clearly showing a rapid depletion of ATP and energy charge in the cell.

A major advantage of the Nash Equilibrium modeling of metabolic pathways is that standard state Gibbs free energies and enthalpies of formation are the key parameters used in the model, which is dramatically fewer compared to traditional Michaelis–Menten models which require an impractical number of kinetic constants in the context of an organ metabolism. In this sense, the NE model can be considered close to a first principles model. To ensure that the predictions of the model were robust against such literature thermodynamic data, a numerical study of parameter uncertainties was conducted. Results showed that percent variances associated with model parameter uncertainties were less than 0.1%. In addition, it was shown that inter-pathway transport fluxes control the convergence of the NE algorithm and that convergence was fast (i.e., under 10 iterations) requiring less than 1 CPU second of computer time. Overall, the minimal input parameter requirement and absence of any major sensitivity to parameter uncertainty are very attractive qualities for NE metabolic modeling, as opposed to those that require extensive reaction kinetic information which is difficult to gather for many biological systems. Similarly, the computational efficiency makes the model very practical for expanding to large problems and more complete metabolic modeling efforts.

## Supplementary Information

Supplementary Information.

## Data Availability

Input and output data from all numerical simulations and plots presented in this paper are available by contacting the corresponding author.
