# Supplementary material for: Modeling energy depletion in rat livers using Nash equilibrium metabolic pathway analysis
Source: Sci Rep. 2022 Mar 3;12:3496. doi: 10.1038/s41598-022-06966-2 (PMC8894355; doi:10.1038/s41598-022-06966-2)
Supplement: Supplementary file 1 — Supplementary Information. [file 41598_2022_6966_MOESM1_ESM.docx]

**Supplemental Material**

The supplemental material consists of a description of ATP content, time dependent behavior of the Nash Equilibrium approach, and values of $\Delta G_{f}^{0}$ and their standard deviations.

**Appendix A: ATP Content**

A number of authors^35,36^ have shown that ATP in SCS correlates with graft viability. However, different publications report ATP content in different units (e.g., pmol/mg protein^33^, μmol/g protein^34^, etc.). Here it shown how to convert from various units of ATP content to ATP concentration.

A typical fresh rat hepatocyte cell contains 985 $\frac{pg protein}{cell}$($\frac{1 mg}{{10}^{9}pg})=9.85x{10}^{-7}\frac{mg protein}{cell}.$Therefore, given a value of ATP content, say $z$, in units of $\frac{pmol}{mg protein}$, the ATP content in $\frac{pmol}{cell}$ is $(9.85x{10}^{-7}\frac{mg protein}{cell})z$.

The average volume of rat hepatocyte cells is 8.354x10^-12^ $\frac{L}{\mathrm{cell}}$. See Gat-Yablonsky et al.^38^. Using the value of (1.1970x10^13^ $\frac{cells}{L})$, the ATP concentration, $c$, in units of mM is given by the equation

$c=$(1.1970x10^13^ $\frac{cells}{L})\left( 9.85x{10}^{-7}\frac{mg protein}{cell} \right)\left( 1x{10}^{-12} \frac{mol}{pmol} \right)z$

$=11.970\left( 9.85x{10}^{-7}\frac{mg protein}{cell} \right)z=1.1790x{10}^{-5}z$.

**Appendix B: The Time Dependent Behavior of Nash Equilibrium**

The key to understanding the connection between Nash Equilibrium (NE) iterations and elapsed time rest on the fact cumulative elapsed time, $t$, is equal to $k\Delta t$, where $k$ is an NE iteration number and $\Delta t$ is a discrete time step.

To begin, transforming any ordinary differential equation (ODE) into an iterative map is accomplished by applying any integration algorithm (i.e., forward or backward Euler, trapezoidal rule, etc.) to the ODE. For example, given

$\frac{dx}{dt}=f(x,t)$ (B1)

applying forward Euler integration gives the iterative map

$x_{k+1}= x_{k}+\frac{dx_{k}}{dt}\Delta t \to x_{k+1}= x_{k}+f(x_{k},t)\Delta t$ (B2)

where $f\left( x,t \right)$ is the right hand side of the ODE. Reversing the process, (i.e., transforming an iterative map into time domain dynamics) is accomplished by arranging the iterations for any given variable as shown in Table 1.

**Table 1: Iterations for Any Variable**

| **iteration** | $\boldsymbol{x}_{\boldsymbol{k}}$ |
| --- | --- |
| 0 | $x_{0}$ |
| 1 | $x_{1}$ |
| 2 | $x_{2}$ |
| . | . |
| . | . |
| . | . |
| $N$ | $x_{N}$ |

Without sacrificing the first principles on which the Nash Equilibrium (NE) approach is built, discrete NE iteration variables can be expressed as functions of time by fitting NE simulation data to continuous functions. Remember, the NE data represents discrete time data because $t=k\Delta t, k=0, 1, 2, \ldots,N$.

Example: ATP Hydrolysis vs. Time. Consider the Nash Equilibrium outer loop iterations for $[ATP]$ and $[ADP]$ content for the liver model in Fig. 2 during static cold storage shown in Table 2. The reason we have chosen ATP and ADP as examples is because liver viability has been strongly correlated to ATP content and ATP is involved in several pathways in the liver model shown in Fig. 2. However, any of the 295 metabolites and cofactors in the liver model could have been selected for analysis.

In static cold storage, ATP is depleted and therefore ATP undergoes hydrolysis, consuming water and producing ADP, Pi, and hydrogen ions in the process. In addition, it is well known that ATP hydrolysis has a sigmoidal response driven by the enzyme ATPase^39^. We will return to this fact later in this appendix.

**Table 2: NE Iterations for** $\boldsymbol{[ATP]}$, $\boldsymbol{[ADP]}$ **&** $\boldsymbol{[}\boldsymbol{H}^{\boldsymbol{+}}\boldsymbol{]}$ **in Static Cold Storage**

| **iteration** | $\boldsymbol{[ATP]}$ **(mM)** | $\boldsymbol{[ADP]}$ **(mM)** | $\boldsymbol{[}\boldsymbol{H}^{\boldsymbol{+}}\boldsymbol{]}$ |
| --- | --- | --- | --- |
| 0 | 7.92338 | 4.67605 | 1.75071x10^-8^ |
| 1 | 5.12754 | 5.42549 | 1.97987x10^-8^ |
| 2 | 1.66728 | 6.04022 | 1.82215x10^-8^ |
| 3 | 0.97670 | 7.58156 | 5.37378x10^-9^ |
| 4 | 0.96420 | 7.04381 | 5.10395x10^-9^ |
| 5 | 0.85790 | 6.95691 | 5.10106x10^-9^ |
| 6 | 0.63436 | 7.06980 | 4.83393x10^-9^ |

Continuous approximations for $\left[ ATP \right]$ and $\left[ ADP \right]$ were generated by least-squares fits of the Nash Equilibrium static cold storage simulation data shown in Table 2 and are given by

$\left[ ATP \right]=e^{(-1.098\log_{10} t+1.477)}$ (B3)

$\left[ ADP \right]=4.711-0.2684t+1.107t^{2}-0.3282t^{3}+0.02702t^{4}$ (B4)

where $t=k\Delta t$ can be viewed as an artificial integration variable. In Eqs. B3 and B4, $\left[ ATP \right]$ and $\left[ ADP \right]$ have $R^{2}$ goodness of fits of 0.9808 and 0.9445 respectively. Differentiating Eq. B3 gives the evolution of $\left[ ATP \right]$ given by

$\frac{d[ATP]}{dt}= -[\frac{1.098}{(ln 10)t}]e^{(-1.098\log_{10} t+1.477)}$ (B5)

Differentiating Eq. B4 and setting the first derivative gives the cubic polynomial

$\frac{d[ADP]}{dt}=-0.2684+1.107t-0.3282t^{2}+0.02702t^{3}$ (B6)

Setting Eq. B6 equal to zero yields only one maximum velocity (reaction rate), $\nu_{max}$, equal to 1.0852 mM/unit time at $t=2\Delta t$ in the interval $k\Delta t=[0, 6\Delta t]$.

Michaelis-Menten Kinetics

If we assume the reaction rate for $\left[ ADP \right]$ is described by Michaelis-Menten kinetics, $K_{M}=1.09409$ mM/unit time, then resulting ODE for $\left[ ADP \right]$ is given by

$\frac{d[ADP]}{dt}=\frac{1.0852\left[ ATP \right]}{1.09409+\left[ ATP \right]}$ (B7)

Equations B5 and B7 form a two-point boundary condition since the initial and final conditions are known. Using the initial conditions at iteration $0$ in Table 2, were integrated to equilibrium using backward Euler integration with a fixed step size of 0.0125 until the dependent variables, $\left[ ATP \right]$ and $\left[ ADP \right]$, most closely match the values of $\left[ ATP \right]$ and $\left[ ADP \right]$ shown in Table 2 for iteration 6. Remember, $t$ is an arbitrary integration variable.

To convert to time, the variable $\Delta t$ can be calculated from

$\Delta t=\frac{\chi}{N}$ (B8)

where $N$ is the total number of NE iterations and where $\chi$ is the value of the independent integration variable at that most closely matches the final conditions. For this illustrative example $\chi=5.618$ and $N=6$ from Table 2; therefore from Eq. B8, $\Delta t=0.9364$ in units of hours.


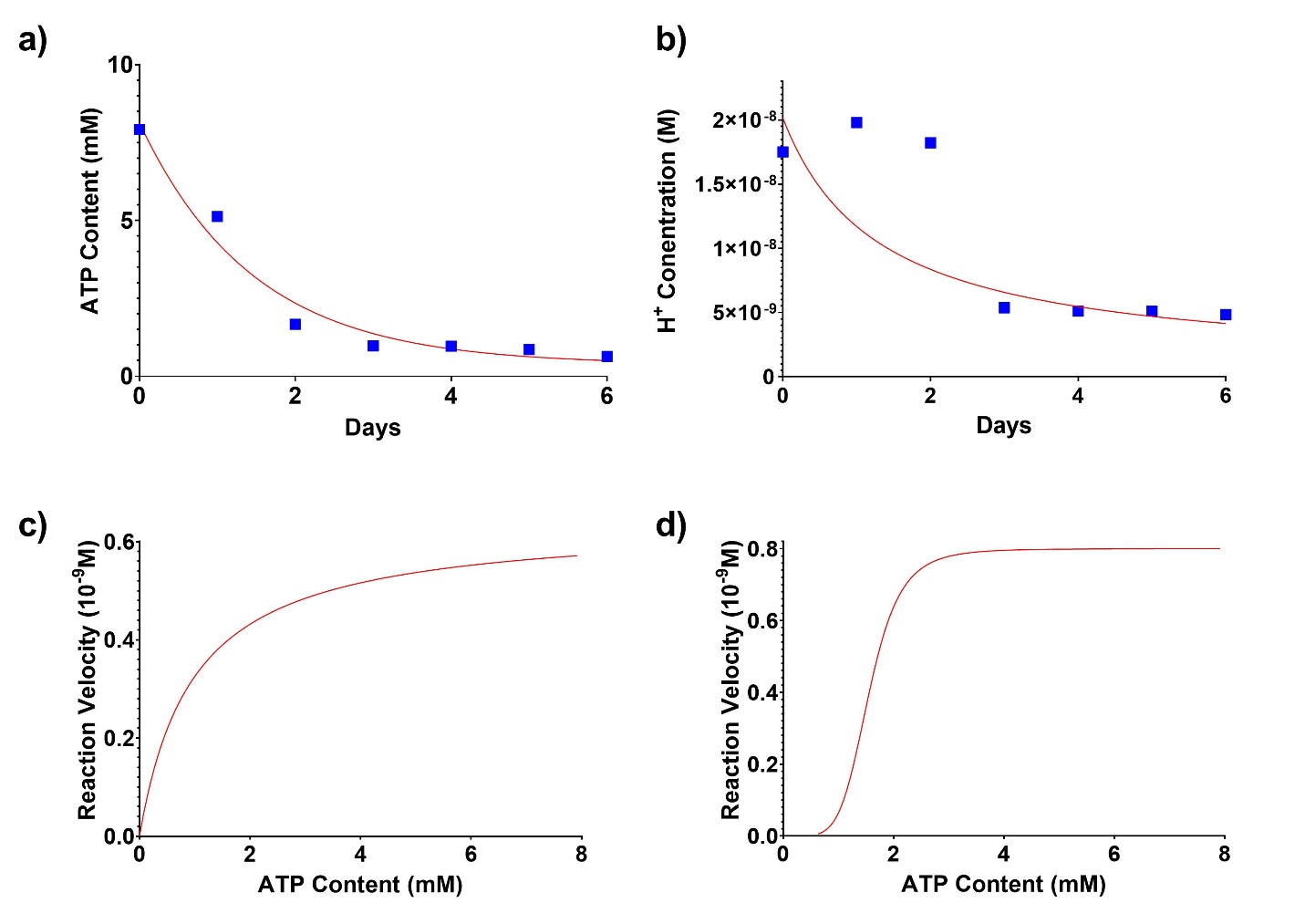


**Figure B: Time dependence of ATP and H^+^ concentrations and reaction velocities for Michaelis-Menten and sigmoidal kinetics for liver model in static cold storage. a) ATP content (mM) vs. time, b) H^+^ concentration (mM) vs. time, c) reaction velocity (**$\boldsymbol{\nu}_{\boldsymbol{H+}}\boldsymbol{)}$ **vs. [ATP] using Michaelis-Menten kinetics,**

**d) reaction velocity (**$\boldsymbol{\nu}_{\boldsymbol{H+}}\boldsymbol{)}$ **vs. [ATP] with sigmoidal kinetics.** **fitted NE simulation data, discrete NE simulation data.**

We could just as easily integrate the following Michaelis-Menten expression for $\frac{d[H^{+}]}{dt}$

$\frac{d[H^{+}]}{dt}=\frac{-6.41048x{10}^{-9}\left[ ATP \right]}{0.96945+\left[ ATP \right]}$ (B9)

with Eq. B5 to determine $\chi$ in Eq. B8. This, in turn, resulted in a value of $\Delta t=0.9385$ hr.

Sigmoidal Kinetics

It is well known that the reaction velocities for ADP, Pi and H^+^ ions as a function of ATP concentration in ATP hydrolysis involve allosteric interactions and can be adequately described by sigmoidal kinetics^39^. In fact, the plots of the NE simulation data for $\left[ ADP \right]$ and pH shown in Figs. 3b) and 4d) respectively both show the signature of sigmoidal kinetics (i.e., the responses of $\left[ ADP \right]$, $\left[ H^{+} \right]$, and pH lag behind the dynamics of $\left[ ATP \right])$.

Sigmoid functions are also known as logistic functions and there are existing models for generalized logistic functions. See Rzadkowski and Sobcak^37^. Other models for sigmoidal kinetics, like that by Hill^38^, use a modified Michaelis-Menten functional form to incorporate cooperativity. In this work, we take a different approach. We use the time dependent function

$y=y\left( 0 \right)+c\ln(1+e^{s(t_{f}-2t-t_{d})})$ (B10)

as a model for sigmoidal kinetics, where $y$ is a dependent variable, $c$ is a constant that accounts for the scale and units of the dependent variable, $s$ is a parameter that controls the shape of the sigmoidal function, $t$ is time in hours, and $t_{f}$ and $t_{d}$ denote the final time and time delay for the NE iterations, which are readily available from NE simulation data.

The derivative of Eq. B10 gives the reaction velocity

$\frac{dy}{dt}=\frac{-2cs}{\left( 1+e^{-s\left( t_{f}-2t-3 \right)} \right)}$ (B11)

Panels b), c) and d) in Fig. B are plots of the $\left[ H^{+} \right]$ vs. time (Eq. B10) and the reaction velocities (rates of consumption) of $y=\left[ H^{+} \right]$ ions for Michaelis-Menten (Eq. B9) and sigmoidal (Eq. B11) kinetics respectively. For Michaelis-Menten kinetics, values of $\nu_{max}=-6.41048x{10}^{-9}$ M/hr. and $K_{M}=0.96945$ mM were determined from the simulation data in Table 2. For sigmoidal kinetics, $y\left( 0 \right)=4.83393x{10}^{-9}$ M, $t_{f}=6$ hr. and $t_{d}=3$ hr. from the simulation data in Table 2, a value of $c=1x{10}^{-8}$ M and an optimal value of $s=0.5$, which was determined by fitting Eq. B10 to the $\left[ H^{+} \right]$ ion NE simulation data shown in Table 2. Also, the value of $c$ was determined using the simulation data in Table 2, where it can be seen that there is an abrupt change in $\left[ H^{+} \right]$ at iteration 3. From Eq. B11, the reaction velocity of $\left[ H^{+} \right]$ is

$\frac{d[H^{+}]}{dt}=\frac{-1x{10}^{-8}}{(1+e^{6\left( t+1 \right)})}$ (B12)

Note that reaction velocity of $\left[ H^{+} \right]$ ions shown in panel d) of Fig. B follow sigmoidal kinetics, are quite different than those for Michaelis-Menten kinetics shown in panel c) and shows that the rate of consumption of $\left[ H^{+} \right]$ ions ‘lags’ behind the behavior of other metabolites. This fact is also consistent with the behavior of pH shown in Fig. 4d).

To represent sigmoidal kinetic in terms of $\left[ ATP \right]$, $t$ in Eq. B12 must be expressed in terms of $\left[ ATP \right]$. To do this we male use of Eq. B3. In particular, taking the natural log of both sides of Eq. B3 and using the relationship between natural log and log base 10, it follows that

$\ln\left[ ATP \right]=-1.098\log_{10} t+1.477=2.303\log_{10} [ATP]$ (B13)

Solving Eq. B13 for $\log_{10} t$ gives the expression

$\log_{10} t=-\left( \frac{2.303}{1.098} \right)\log_{10} \left[ ATP \right]+\frac{1.477}{1.098}=-2.097\log_{10} \left[ ATP \right]+1.345$ (B14)

Raising both sides of Eq. B14 to the power 10 and using the properties of exponentials (i.e., ${10}^{(a+b)}={10}^{a}{10}^{b}$ and ${10}^{ab}={(10}^{a})^{b}$) we have that

$t={10}^{1.345-2.097\log_{10} \left[ ATP \right]}={10}^{1.345}{10}^{-2.097\log_{10} \left[ ATP \right]}=\frac{22.13}{{10}^{2.097\log_{10} \left[ ATP \right]}}=\frac{22.13}{[ATP]^{2.097}}$ (B15)

Using Eq. B15 in Eq. B12 gives

$\frac{d[H^{+}]}{dt}=[\frac{-1x{10}^{-8}}{\left( 1+e^{6\left( \frac{22.13}{[ATP]^{2.097}}+1 \right)} \right)}]$ (B16)

which is an approximate sigmoidal kinetics expression for the rate of consumption of $[H^{+}]$ since Eq. B3 is a fit of the NE simulation data.

Finally, while we have used both Michaelis-Menten and sigmoidal kinetics to describe the overall behavior of the reaction velocity of $\left[ H^{+} \right]$ ions for the liver model in static cold storage, other kinetic models for other metabolites and/or cofactors could be proposed and analyzed in the very same way.

**Appendix C****: Values of** $\boldsymbol{\Delta}\boldsymbol{G}_{\boldsymbol{f}}^{\boldsymbol{0}}$ **and Their Standard Deviations**

The data shown in this appendix was taken from the eQuilibrator database (<http://equilibrator.weizmann.ac.il/>) and was printed directly from the Nash Equilibrium software.
